# Supplementary material for: Functional divergence of plant SCAR/WAVE proteins is determined by intrinsically disordered regions
Source: Sci Adv. 2025 May 21;11(21):eadt6107. doi: 10.1126/sciadv.adt6107 (PMC12094195; doi:10.1126/sciadv.adt6107)
Supplement: Supplementary file 1 — Figs. S1 to S10 Tables S1 to S4 Legend for data S1 [file sciadv.adt6107_sm.pdf]

Science Advances
AAAS

Supplementary Materials for

Functional divergence of plant SCAR/WAVE proteins is determined by intrinsically disordered regions

Sabine Brumm *et al.*

Corresponding author: Sebastian Schornack, sebastian.schornack@slcu.cam.ac.uk

Sci. Adv. **11**, eadt6107 (2025)
DOI: 10.1126/sciadv.adt6107

The PDF file includes:

Figs. S1 to S10
Tables S1 to S4
Legend for data S1

Other Supplementary Material for this manuscript includes the following:

Data S1

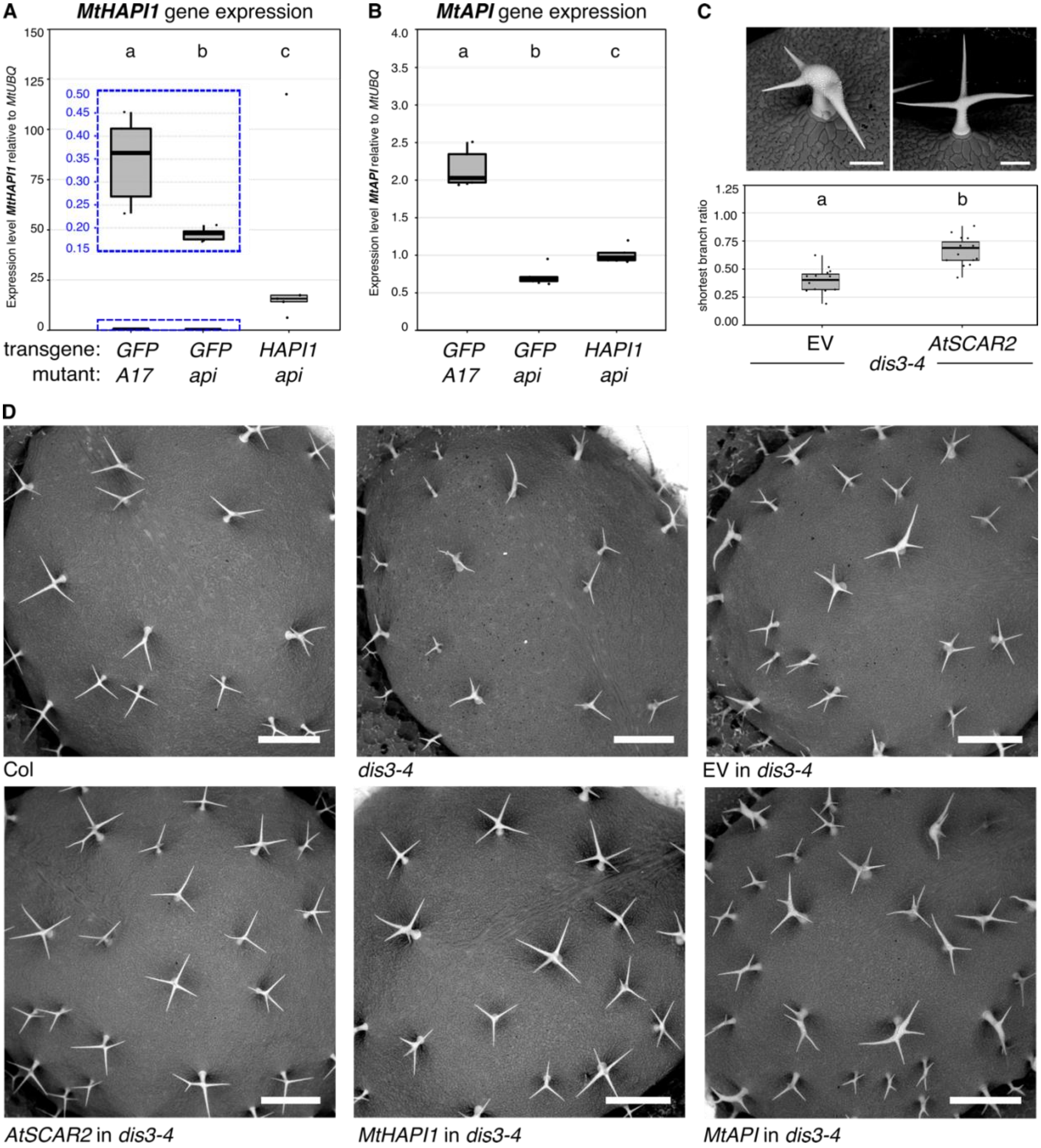

Fig. S1: Expression levels of *MtHAPI1* in transgenic Medicago roots and *MtHAPI1* and MtAPI complementation assays in *A. thaliana dis3-4* mutants.

(A-B) Quantification of *MtHAPI1* (A) and *MtAPI* (B) mRNA in independently transformed *A17* and *api M. truncatula* roots (n=5). MtUBQ was used as reference gene in the 2-ΔΔCP calculations. GFP was used as control. Statistics: Shapiro-Wilk test, followed by Kruskal-Wallis with Bonferroni correction; significance groups: a, b and c. (**C**) Controls for the trichome branch length rescue in *A. thaliana dis3-4* (Fig1): *EV* (negative) and *AtSCAR2* (positive). Scale bars, 90 µm. Shortest branch ratio (n = 15 / genotype). Statistics: Shapiro-Wilk test, followed by Kruskal-Wallis

with Bonferroni p-value adjustment (alpha = 0.05); significance differences are indicated by letters a and b. **(D)** Scanning electron micrographs of *A. thaliana* leaves from untransformed Col, *dis3-4* lines and *dis3-4* lines transformed with *empty vector* (EV), *AtSCAR2*, *MtHAPI1* or *MtAPI* driven by the *AtUBQ3* promoter. Scale bars, 0.5 mm.

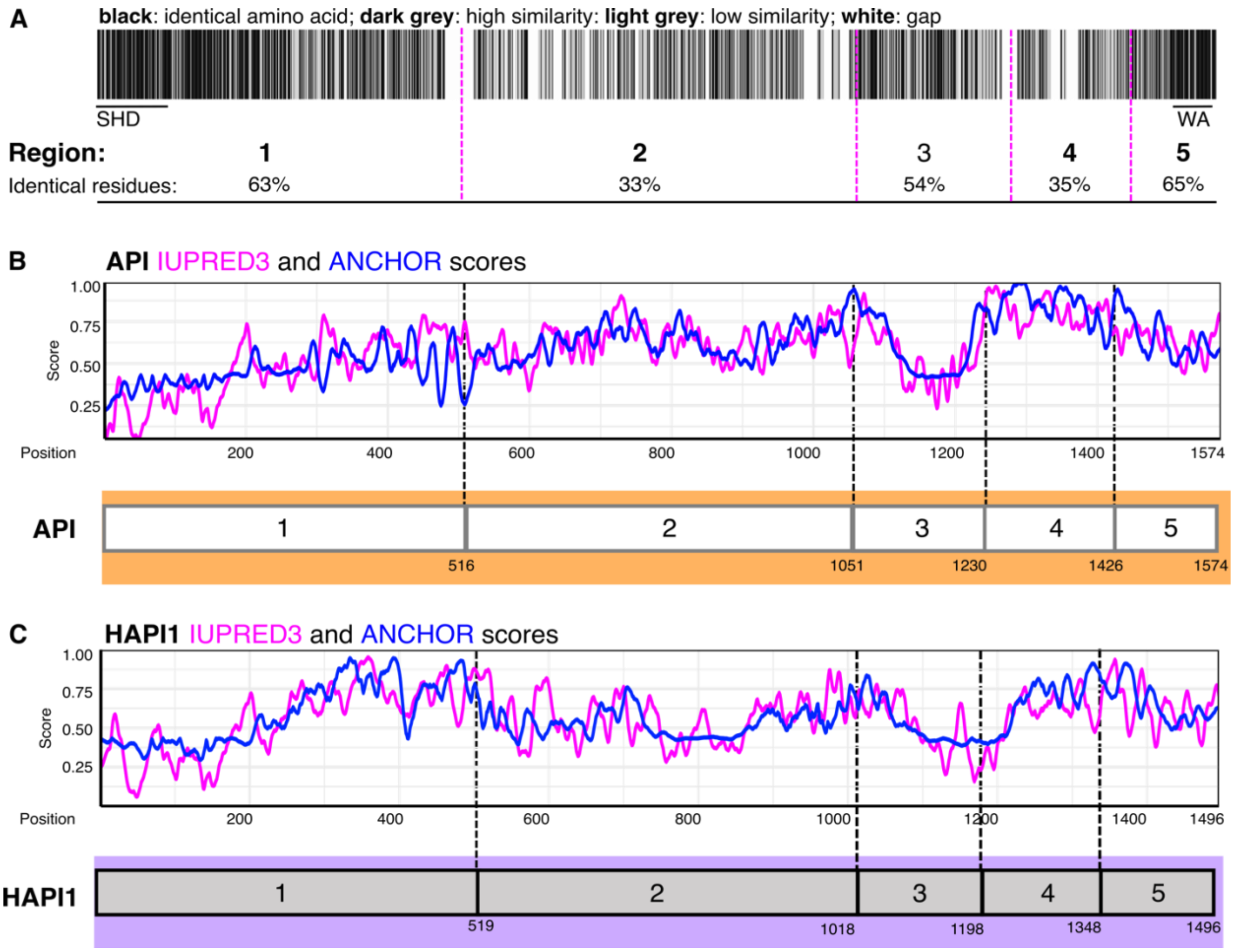

Fig. S2: Bioinformatic analysis of *Mt*API and *Mt*HAPI1 protein sequences
(A) Schematic barcode representation of *Mt*API and *Mt*HAPI1 amino acid conservation. Colours represent identical (black), highly similar (dark grey), moderately similar (light grey) amino acid residues, and alignment gaps (white). (**B** and **C)** Prediction of intrinsically disordered protein regions (IUPRED3, pink) and disordered protein binding regions (ANCHOR2, blue) by IUPRED3 in *Mt*API (B) and *Mt*HAPI1 (C).

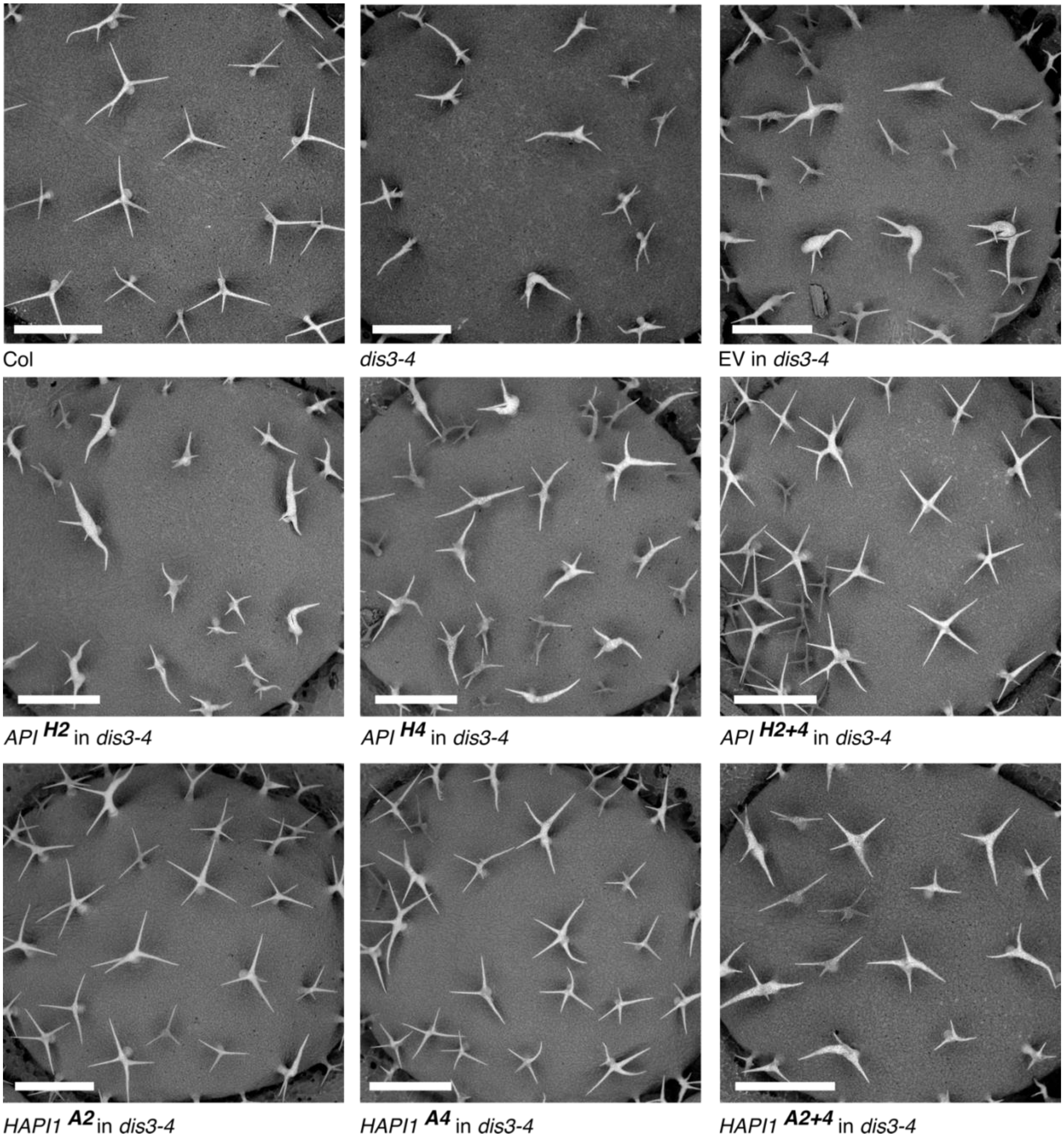

Fig. S3: *A. thaliana* complementation studies with chimeric proteins.
Scanning electron micrographs of *A. thaliana* leaves from Col, *dis3-4*, and *dis3-4* lines transformed with *empty vector* (EV) and chimeric *API/HAPI1* variants under the *AtUBQ3* promoter. Scale bars, 0.5 mm.

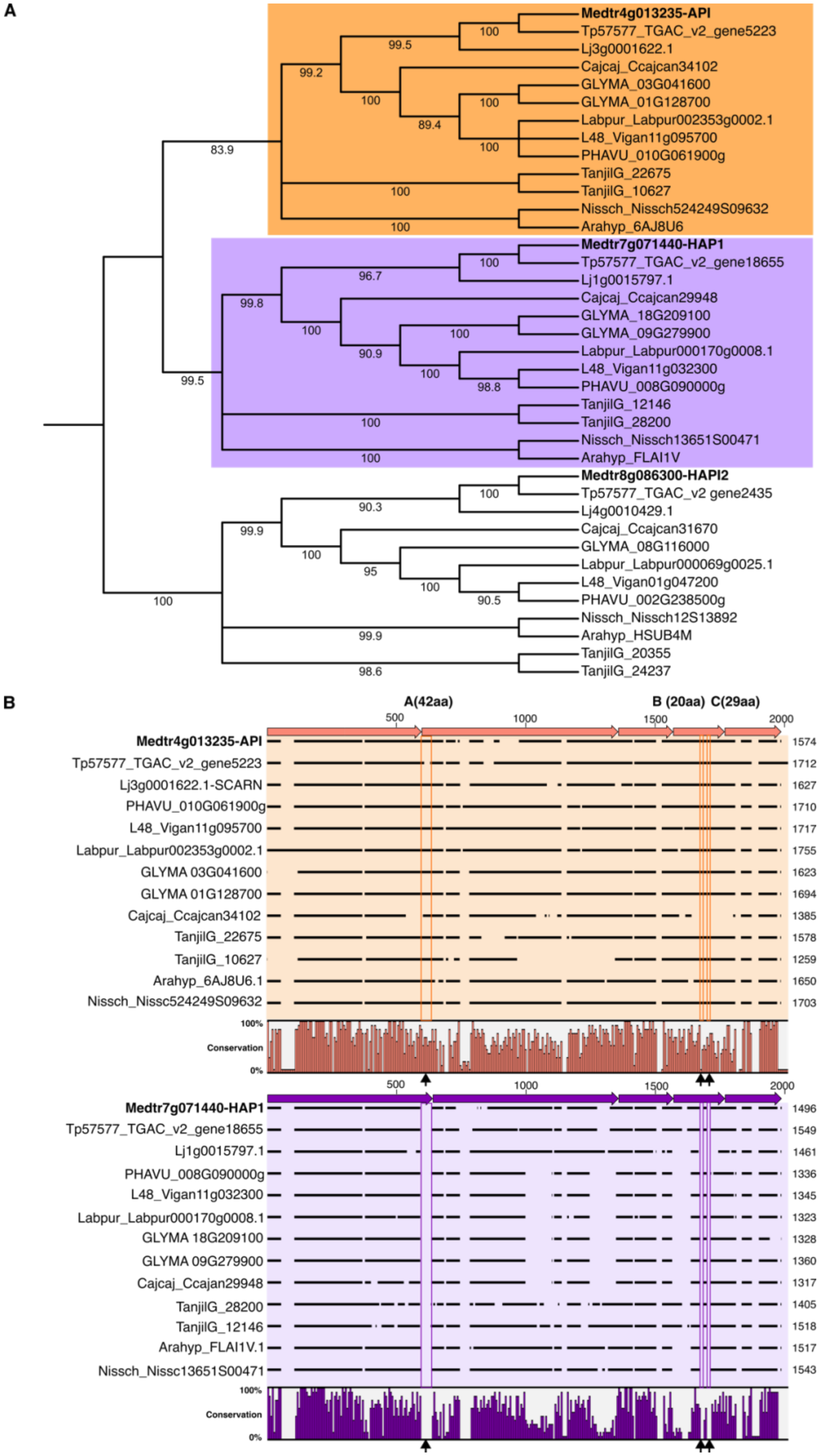

Fig. S4: Sequence conservation analysis of *Mt*API and *Mt*HAPI1.

(A) Maximum likelihood phylogenetic tree of SCAR proteins from various legume species, showing three distinct clades. *Mt*API (orange) and *Mt*HAPI1 (purple) like proteins are more closely related to each other than to *Mt*HAPI2-like proteins. Node bootstrap values are indicated. Species abbreviations: Medtr (*Medicago truncatula*), Lj (*Lotus japonicus MG20*), TanjilG (*Lupinus angustifolius*), LR48_Vigan (*Vigna angularis*), PHAVU (*Phaseolus vulgaris*), Tp (*Trifolium pratense*), GLYMA (*Glycine max*), Nissch (*Nissolia schottii*), Arahyp_arahy (*Arachis hypogaea*), Cajcaj_Ccajan (*Cajanus cajan*), Labpur (*Lablab purpureus*). **(B)** Sequence conservation analysis of *Mt*API and *Mt*HAPI1-like legume proteins identifies three unique amino acid segments in *Mt*API-like sequences (orange boxes over black arrowheads) absent in *Mt*HAPI1-like sequences (purple boxes over black arrowheads). The five regions of varying conservation are indicated by orange and purple arrows. Black lines represent aligned amino acids, while white spaces indicate alignment gaps. The total numbers of amino acids per sequence are shown at the end of each alignment. Histograms below the alignments show the percentage of conservation for each position (orange bars for *Mt*API-like, purple bars for *Mt*HAPI1-like sequences).

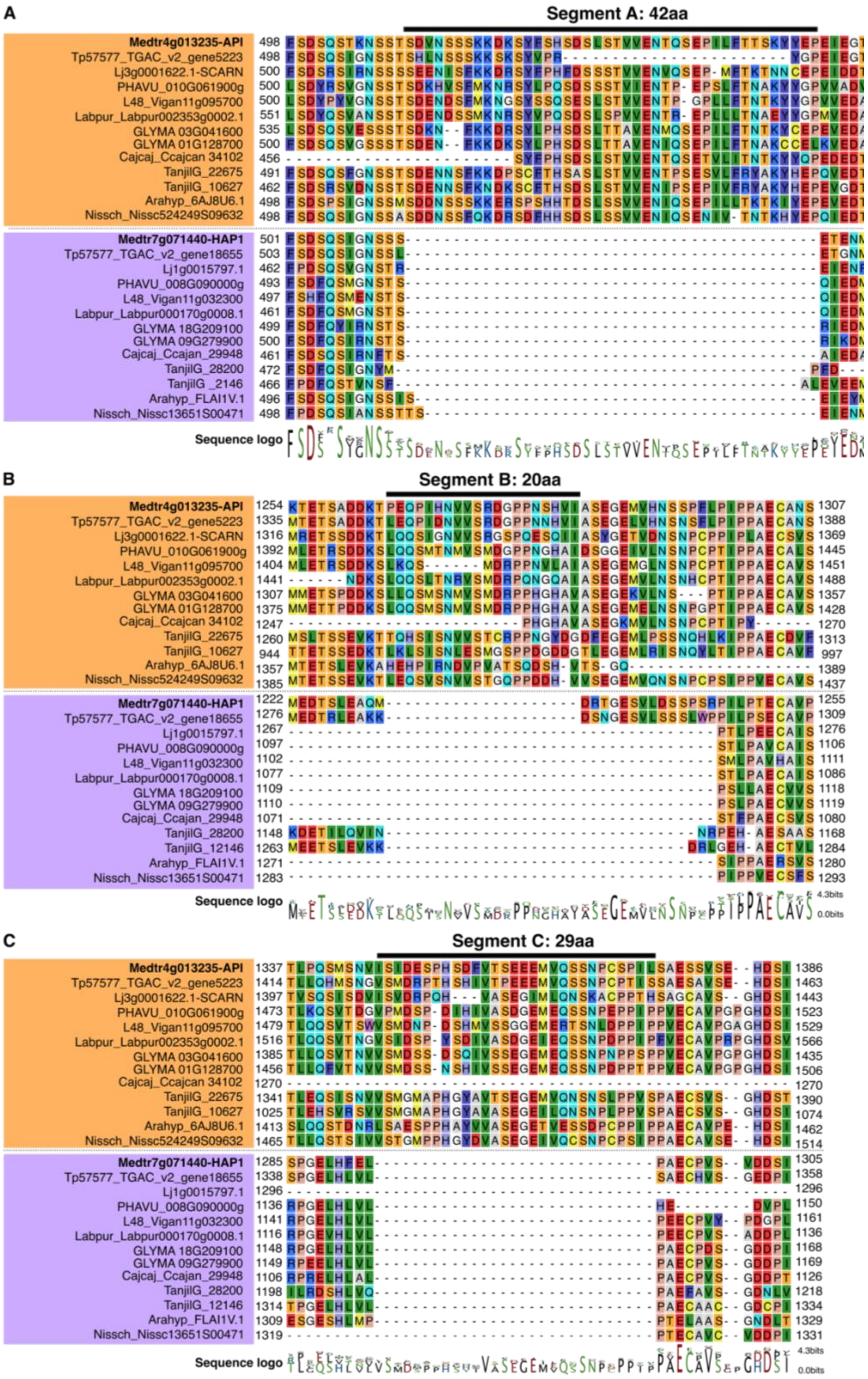

Fig. S5: Detailed alignment of *Mt*API segments A, B, and C in closely related legume homologs.

(**A** to **C**) Detailed sections of the alignment from fig. S4, focusing on *Mt*API segments A (A), B (B), and C (C). Amino acid positions at the start and end of each alignment are indicated. The backgrounds of amino acids are coloured according to the RasMol colour scheme. Orange: *Mt*API-like sequences; Purple: *Mt*HAPI1-like sequences. Segments A, B, and C are highlighted by black lines above each alignment.

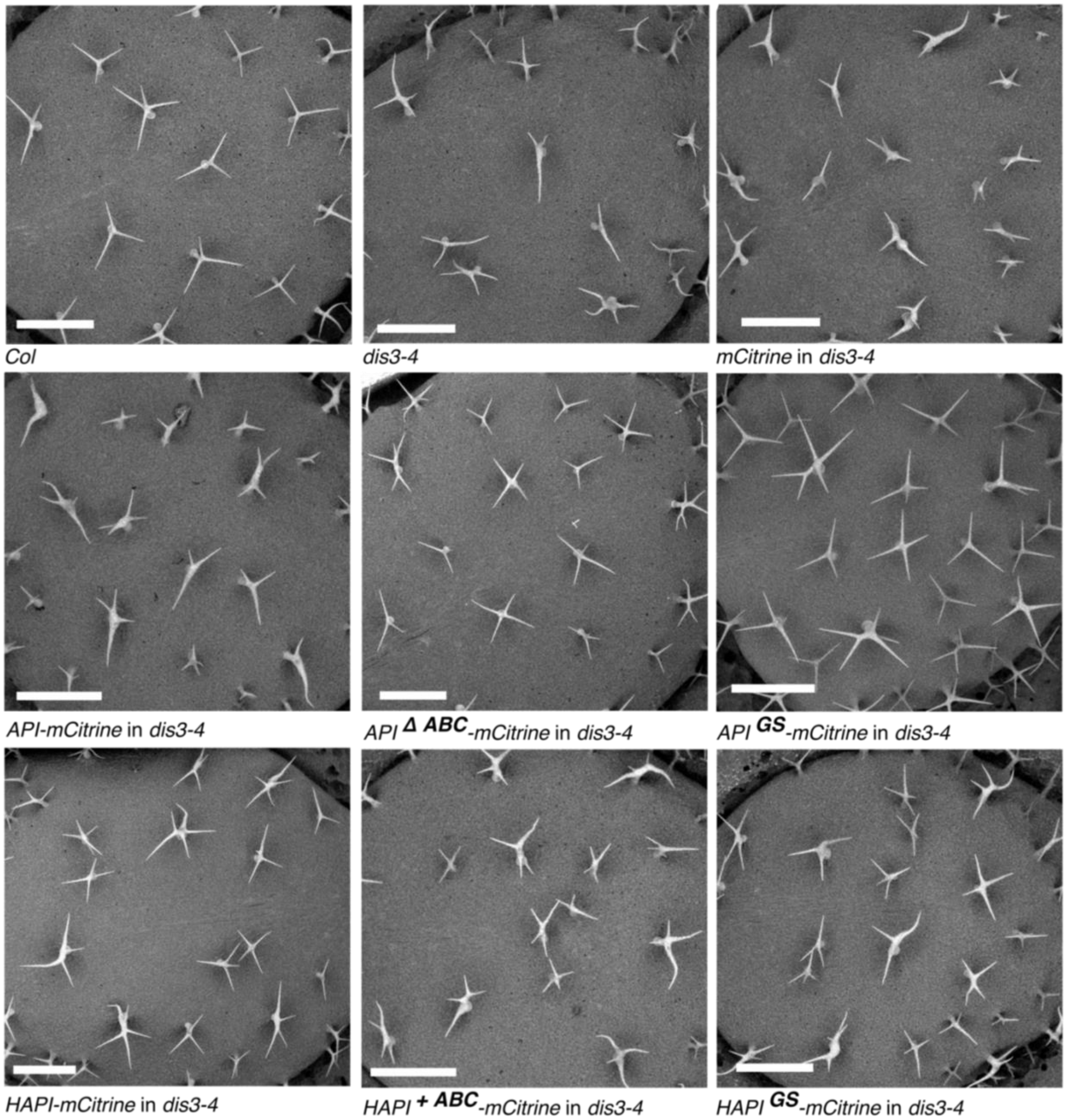

Fig. S6: *A. thaliana* leaf overview from complementation studies with MtAPI and MtHAPI1 segment A, B, C, and GS-linker variants.
Scanning electron micrographs of *A. thaliana* leaves from Col, *dis3-4*, and *dis3-4* lines expressing either an empty vector (EV) or mutant *MtAPI* or *MtHAPI1* variants (segments A, B, C, and GS-linkers) under the *AtUBQ3* promoter. Scale bars, 0.5 mm.

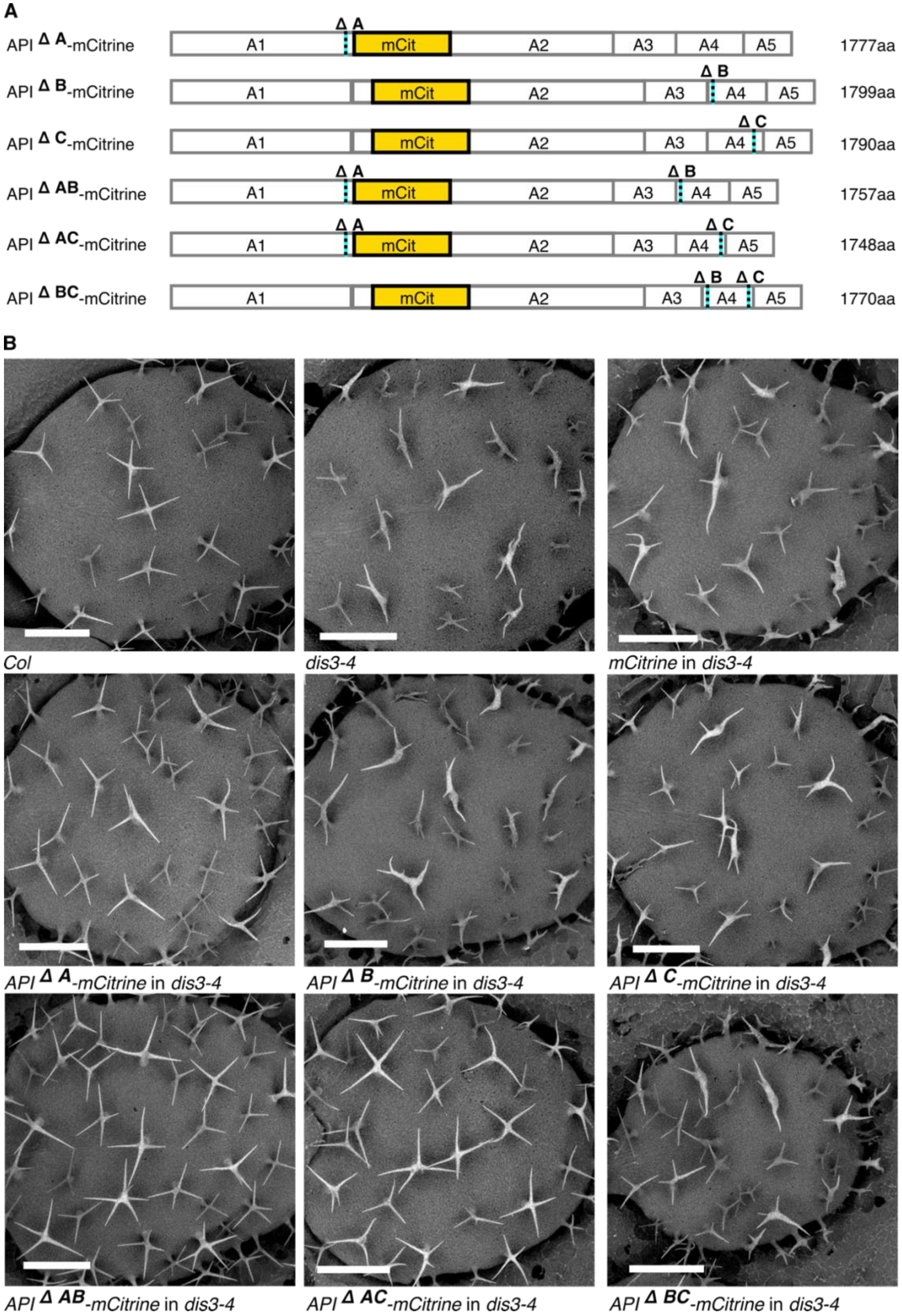

Fig. S7: Impact of segment A on *Mt*API function in *A. thaliana* trichome development.

(**A)** Schematic of *Mt*API protein variants with single or double deletions in segments A, B, and C (dotted lines over cyan). mCitrine tags are shown in yellow, with protein size changes depicted. (**B**) Scanning electron micrographs of *A. thaliana* leaves from Col, *dis3-4*, and *dis3-4* lines either expressing empty vector (EV) or *Mt*API segment A, B and C deletion variants under the *AtUBQ3* promoter. Scale bars, 0.5 mm.

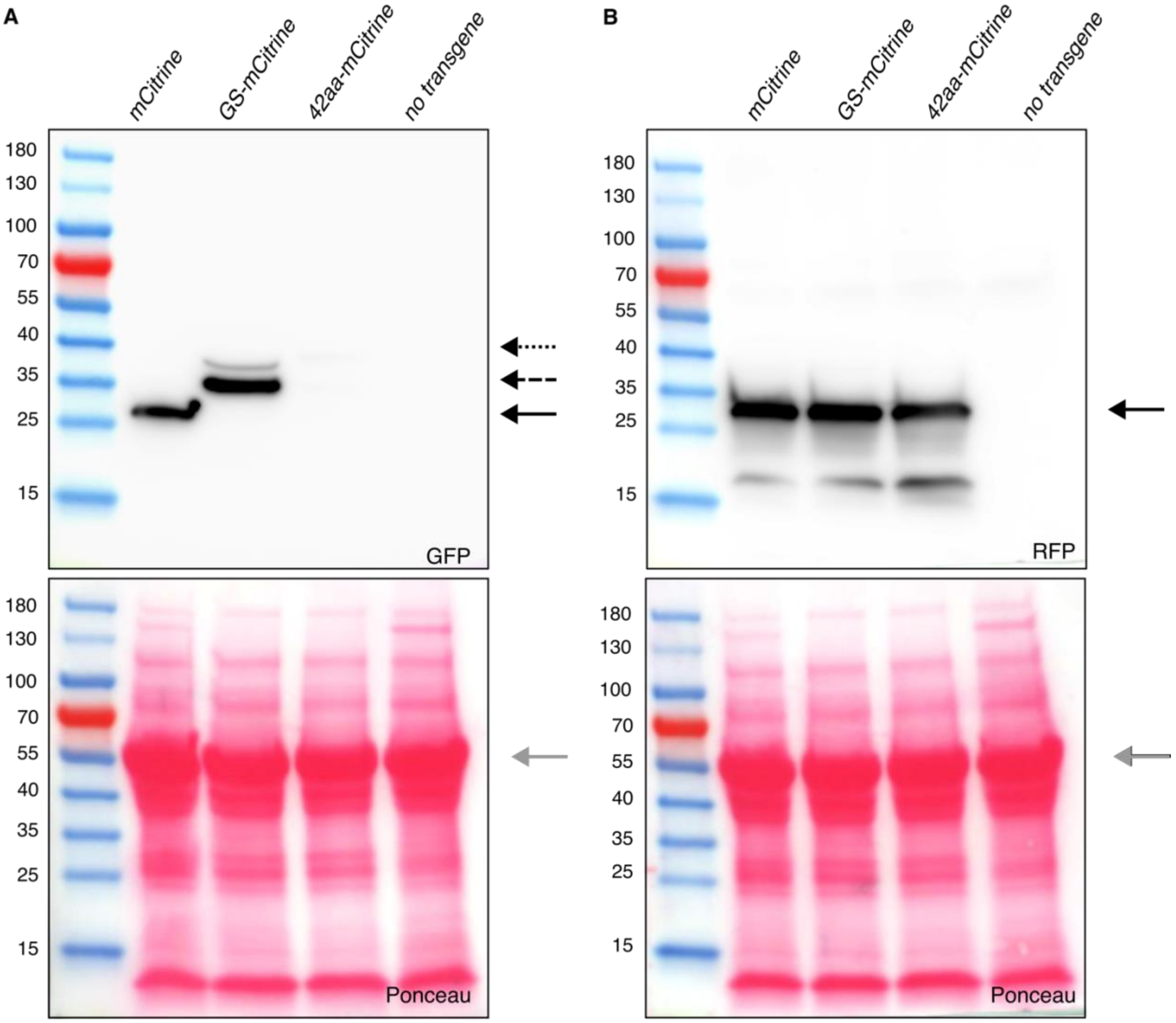

Fig. S8: Western Blot analysis of segment A expression in N. benthamiana leaves
(A and B) Western Blot analysis of mCitrine, GS-mCitrine and 42aa-mCitrine along with coexpressed dsRed, in transiently transformed *N. benthamiana* leaves. dsRed serves as positive control for transformation, while a sample from untransformed leaves (no transgene) serves as negative control. Samples were simultaneously run on two gels, membranes stained with Ponceau, and probed either with GFP (A) or RFP (B) antibodies. Arrows indicate expected sizes of protein band: Rubisco (55kDa, grey), mCitrine (27kDa, black), dsRed (28kDa, black) GS-mCitrine (36kDa, black with long dotted line) and weakly detectable 42aa-mCitrine (38kDa, black with short dotted line). Prestained protein ladder: Page Ruler 10-180kDa.

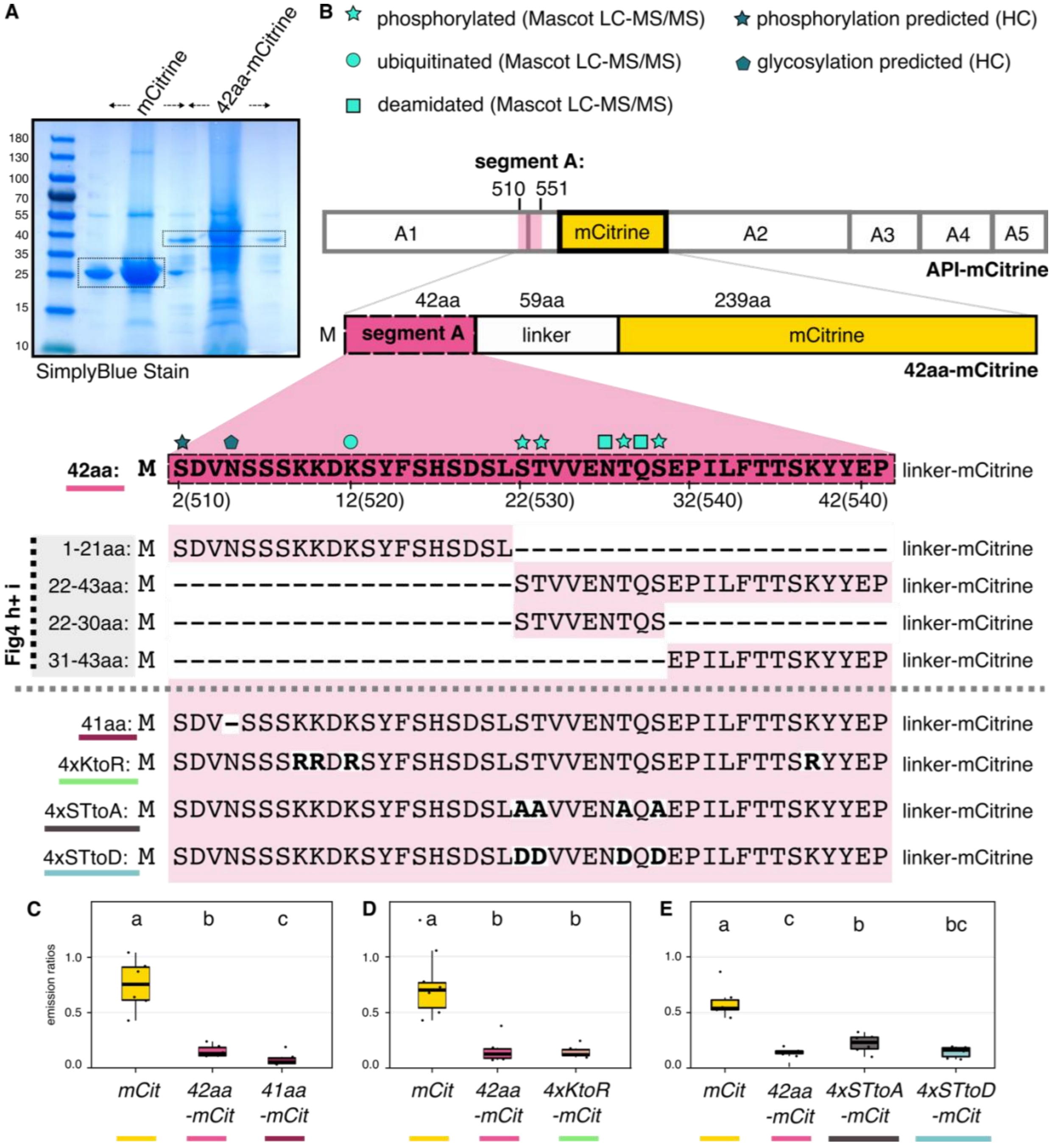

Fig. S9: Post translational modification analysis of segment A.
(**A**) SDS gel of immunoprecipitated mCitrine and 42aa-mCitrine proteins stained with SimplyBlue. Arrows indicated spillovers. Dotted lines indicate the gel sections used for LC-MS/MS analysis and MASCOT search, whose results guided the design of the subsequent 42aa-mCitrine deletion and substitution constructs. (**B**) Schematic of API-mCitrine, 42aa-mCitrine, and derived deletion/substitution constructs. Predicted and experimentally validated post- translational modification (PTM) sites in API segment A are mapped onto the 42-amino acid sequence.

Numbers indicate amino acid positions in 42aa-mCitrine, with API-mCitrine positions in brackets. PMTs were predicted using MusiteDeep (https://www.musite.net/), with high-confidence (HC, score > 0.5) sites indicated by symbols with dark filling. Experimentally confirmed PTMs (via LC-MS/MS) are marked with cyan symbols. PTM types: phosphorylation (star), ubiquitination (circle), deamination (square), glucosylation (pentagon). (**C** to **E**) Quantification of mCitrine fluorescence in 42aa-mCitrine derived constructs relative to dsRed. 41aa-mCitrine (C), 4xKtoR-mCitrine (D), 4xSTtoA-mCitrine and 4xSTtoD-mCitrine (E) ratios compared to mCitrine (mCit) and 42aa-mCitrine controls. mCitrine/dsRed signals were calculated using the FIJI plugin FRETENATOR (n =6). The different constructs are colour coded. Statistics: Shapiro-Wilk test Kruskal-Wallis with Bonferroni correction; Statistics: Shapiro-Wilk test, followed by Kruskal-Wallis with Bonferroni correction; significance difference groups indicated by letters a, b, c.

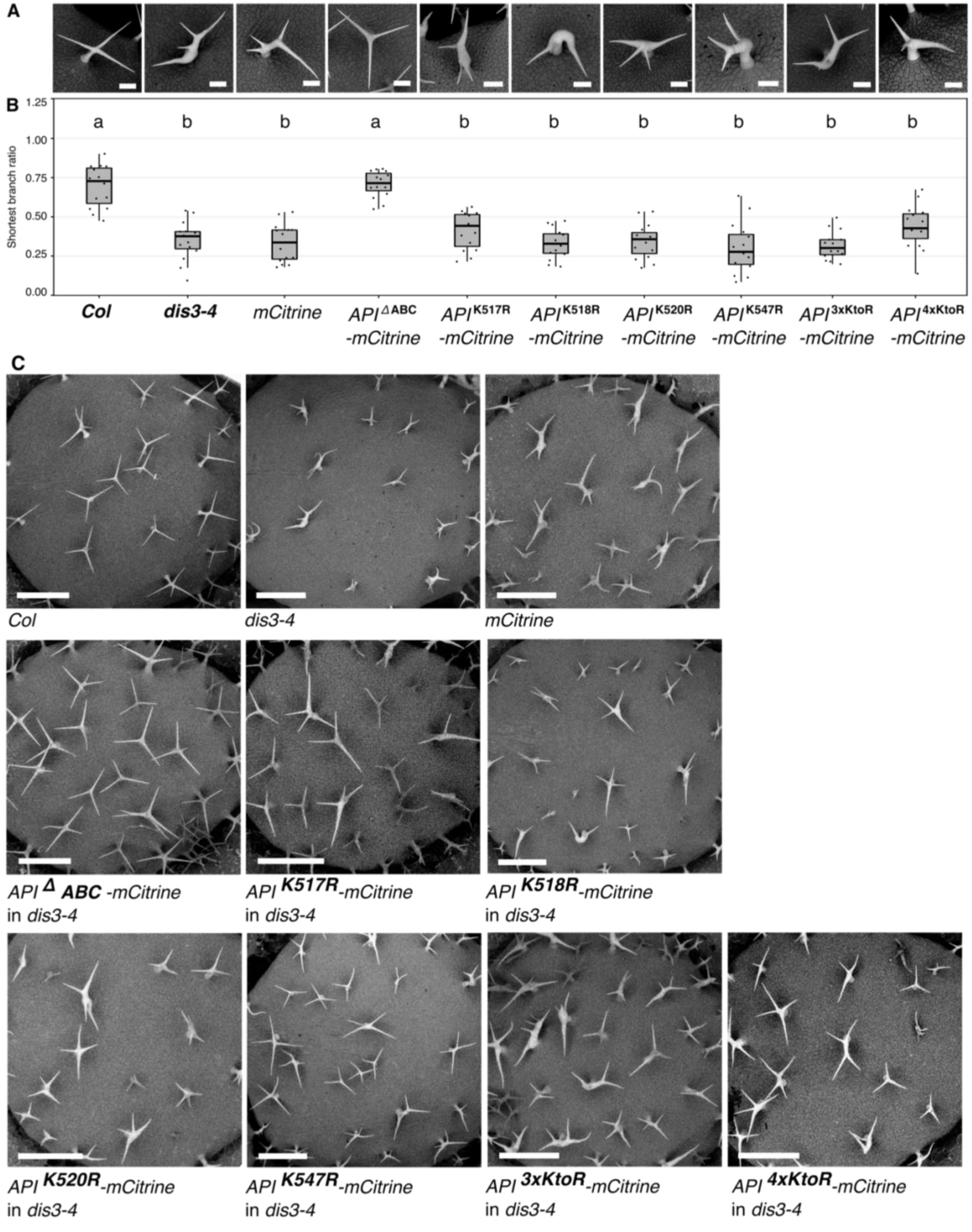

Fig. S10: Expression of API-mCitrine KtoR mutants in *A. thaliana*.

(**A**) Scanning electron micrographs of *A. thaliana* trichomes from Col, *dis3-4*, and *dis3-4* lines expressing mCitrine and *Mt*API segment A lysine substitution variants under the *AtUBQ3* promoter. Scale bars, 90µm. (**B**) Lysine-to-arginine (KtoR) substitutions in *Mt*API segment A do not affect *Mt*API functionality in the *A. thaliana dis3-4* mutant. Shortest trichome branch ratio (n=15/genotype). Statistical significance differences (Shapiro-Wilk test, followed by Kruskal-Wallis with Bonferroni correction) are indicated by letters a and b (**C**) Scanning electron micrographs of *A. thaliana* leaves from trichome magnification in (B). Scale bars, 0.5mm.

Table S1: Primer sequences mentioned in this study

Primer name	Primer sequence
pl_apiSHD_F_AG	gatccaagcttctgcagcccATGCCGATATCGAAGTATC
API1_R_AG	tatggggaagctggttGGAAGAACTATTTACATCTGACG
API2_F_AG	aaatatgtcatccAAGAAAG ATAAATCTTATTTCTCTCATTC
API2_R_AG	gtttcattggatcCACAGCATACTGTGTAGAGTTG
API3.4.5._F_AG	aaagcatgtcatgGATCCATTGAAGTCTCTTCTC
API1.2.3_R_AG	aaacagcacattc AGCAGGTAGTATTGGTGC
API4_HAPI1_5_F_AG	aatactgcctgctGCATATACTCTCTCTGGAGATG
apiMID_hapi1WH2_R_AG	ttggtggtgaAATACCCAATTGTCTCTGC
hapi1MID_apiWH2_F_AG	ctcacctgagTCGCTCATGGTGCCACCA
apiWH2_pl_R_AG	tcgagggtacctctagacccTCAAGAATCACTCCAACTATCCTCATC
pl_hapi1SHD_F_AG	gatccaagcttctgcagcccATGCCTATCTCAAGGTAC
HAPI1_1_R_AG	atttatctttcttGGATGACATATTTTCAGTCTC
HAPI1_2_F_AG	aaatagttcttccAACCAGCTTCCCCATACTG
HAPI1_2_R_AG	acttcaatggatcCATGACATGCTTTGTTGG
HAPI1_3.4.5._F_AG	acagtatgctgtgGATCCAATGAAACCTCTACTTC
HAPI1_1.2.3._R_AG	cagagagagtatatgcAGCAGGCAGTATTTGTGG
HAPI1_4_API5_F_AG	aatactacctgctGAATGTGCTGTTTTTGAGAC
hapi1MID_apiWH2_R_AG	ccatgagcgaCTCAGGTGAGACTAAATGATC
apiMID_hapiWH2_F_AG	attgggtattTCACCACCAAGTACAGAAATTG
hapi1WH2_pl_R_AG	tcgagggtacctctagacccTCAAGAATCACTCCAACTG
SB295	GGGGACAAGTTTGTACaaaaaagcaggctATGTCAGATGTAAATAGTTCTTCCAAG

SB296	GGGGACCACTTTGTAcaagaaagctgggttTCACTTGTACAGCTCGTCCATG
SB323	GGGGACAAGTTTGTACaaaaaagcaggctATGGGAGGTTCTGGTGGAGGTGGATCAG
SB329	GGGGACAAGTTTGTACaaaaaagcaggctATGTCAGATGTAAGTTCTTCCAAGAAAGATAAATC
SB324	GGGGACAAGTTTGTACaaaaaagcaggctATGAGCACTGTTGTTGAAAATACACAATC
SB325	GGGGACAAGTTTGTACaaaaaagcaggctATGGAACCCATTTTATTCACAACTTC
SB343	GGGGACAAGTTTGTACaaaaaagcaggctatgAGCACTGTTGTTGAAAATACACAATCAGAAATTGAAGGTACACCATCGAACC
SB342	GGGGACAAGTTTGTACaaaaaagcaggctATGTCAGATGTAAATAGTTCTTCCAG
SB268	AAGCTGACCCTGAAGTTCATCTGC
SB269	CTTGTAGTTGCCGTCGTCCTTGAA
SB304	AAGGATGCCGTGAAGAAGATGT
SB305	GCATCGTAGTCAGGAGTCAACC
SB306	GGCACTCACAAACGTCTATTTC
SB307	ACCTGGGAGGCATCCTGCTTAT

Table S2: Description of vector and coding sequences mentioned in this study.

Name	Source	Description
pKGW_RR_MGW	Gavrin et al., 2020 (*19*)	Whole vector sequence of multisite Gateway-compatible destination vector with DsRed casette
pENTR4_1_prAtUBQ3p	Gavrin et al., 2020 (*19*)	Whole vector sequence of pENTR vector with Arabidopsis UBQ3 (Gavrin ref) flanked by attL4 and attR1 sites
pENTR4_1_prMtAPI	Gavrin et al., 2020 (*19*)	Whole vector sequence of pENTR vector with 2kb Medicago API promoter (Gavrin ref) flanked by attL4 and attR1 sites
pENTR_p2rp3_T35STerm	Gavrin et al., 2020 (*19*)	Whole vector sequence of pENTR vector with T35S terminator flanked by attR2 and attL3 sites
API	Gavrin et al., 2020 (*19*)	Coding sequence of Medicago API (Medtr4g013235)
HAPI1	Gavrin et al., 2020 (*19*)	Coding sequence of Medicago HAPI1 Medtr7g071440
API_H2	This paper	Coding sequence of API chimera
API_H4	This paper	Coding sequence of API chimera
API_H2+4	This paper	Coding sequence of API chimera
HAPI1_A2	This paper	Coding sequence of HAPI1 chimera
HAPI1_A4	This paper	Coding sequence of HAPI1 chimera
HAPI1_A2+4	This paper	Coding sequence of HAPI1 chimera
API_mCitrine	This paper	Coding sequence of API fusion
API_delABC_mCitrine	This paper	Coding sequence of API variant fusion
API_GS_mCitrine	This paper	Coding sequence of API variant fusion
API_delA_mCitrine	This paper	Coding sequence of API variant fusion
API_delB_mCitrine	This paper	Coding sequence of API variant fusion
API_delC_mCitrine	This paper	Coding sequence of API variant fusion
API_delAB_mCitrine	This paper	Coding sequence of API variant fusion
API_delBC_mCitrine	This paper	Coding sequence of API variant fusion
API_K517R_mCitrine	This paper	Coding sequence of API variant fusion
API_K518R_mCitrine	This paper	Coding sequence of API variant fusion

API_K520R_mCitrine	This paper	Coding sequence of API variant fusion
API_K547R_mCitrine	This paper	Coding sequence of API variant fusion
API_3xKtoR_mCitrine	This paper	Coding sequence of API variant fusion
API_K4xKtoR_mCitrine	This paper	Coding sequence of API variant fusion
HAPI1_mCitrine	This paper	Coding sequence of HAPI1 fusion
HAPI1_insABC_mCitrine	This paper	Coding sequence of HAPI1 variant fusion
HAPI1_GS_mCitrine	This paper	Coding sequence of HAPI1 variant fusion
42aa_mCitrine	This paper	Coding sequence of mCitrine variant fusion
GS_mCitrine	This paper	Coding sequence of mCitrine variant fusion
41aa_mCitrine	This paper	Coding sequence of mCitrine variant fusion
2-20aa_mCitrine	This paper	Coding sequence of mCitrine variant fusion
22_43aa_mCitrine	This paper	Coding sequence of mCitrine variant fusion
31_43aa_mCitrine	This paper	Coding sequence of mCitrine variant fusion
22_30aa-mCitrine	This paper	Coding sequence of mCitrine variant fusion
4xSTtoA_mCitrine	This paper	Coding sequence of mCitrine variant fusion
4xSTtoD_mCitrine	This paper	Coding sequence of mCitrine variant fusion
7xSTtoA_mCitrine	This paper	Coding sequence of mCitrine variant fusion
7xSTtoD_mCitrine	This paper	Coding sequence of mCitrine variant fusion
9xSTYtoA_mCitrine	This paper	Coding sequence of mCitrine variant fusion
9xSTYtoD_mCitrine	This paper	Coding sequence of mCitrine variant fusion
4xKtoR_mCitrine	This paper	Coding sequence of mCitrine variant fusion

Table S3: mCitrine sample protein hits.

Protein hit	Description	Matches
1::sp|cRAP054|P04264|K2C1_HUMAN	Keratin, type II cytoskeletal 1 OS=Homo sapiens GN=KRT1 PE=1 SV=6	63
3::mCitrine		596
3::42aa_linker_mCitrine		576
1::sp|cRAP041|P35527|K1C9_HUMAN	Keratin, type I cytoskeletal 9 OS=Homo sapiens GN=KRT9 PE=1 SV=3	48
1::sp|cRAP032|P42212|GFP_AEQVI	Green fluorescent protein OS=Aequorea victoria GN=GFP PE=1 SV=1	449
1::sp|cRAP053|P35908|K22E_HUMAN	Keratin, type II cytoskeletal 2 epidermal OS=Homo sapiens GN=KRT2 PE=1 SV=2	38
1::sp|cRAP039|P13645|K1C10_HUMAN	Keratin, type I cytoskeletal 10 OS=Homo sapiens GN=KRT10 PE=1 SV=6	39
1::sp|cRAP112|P00761|TRYP_PIG	Trypsin OS=Sus scrofa PE=1 SV=1	90
2::tr|A0A0A8IBT8|A0A0A8IBT8_NICBE	Glyceraldehyde-3-phosphate dehydrogenase OS=Nicotiana benthamiana OX=4100 GN=NbGAPDH-A PE=2 SV=1	13
2::tr|A0A286RNF7|A0A286RNF7_NICBE	Carbonic anhydrase OS=Nicotiana benthamiana OX=4100 PE=2 SV=1	14
2::tr|Q2LAH1|Q2LAH1_NICBE	Chloroplast photosystem II 22 kDa component OS=Nicotiana benthamiana OX=4100 GN=psbS2 PE=4 SV=1	6
2::tr|Q2LAH0|Q2LAH0_NICBE	Chloroplast photosystem II 22 kDa component OS=Nicotiana benthamiana OX=4100 GN=psbS1 PE=2 SV=1	6
2::tr|A4D0J9|A4D0J9_NICBE	Carbonic anhydrase (Fragment) OS=Nicotiana benthamiana OX=4100 PE=2 SV=1	7
2::tr|I0B7J6|I0B7J6_NICBE	Chloroplast PsbP2 OS=Nicotiana benthamiana OX=4100 GN=psbP2 PE=2 SV=1	10
2::tr|E5LLE7|E5LLE7_NICBE	Phosphoglycerate kinase OS=Nicotiana benthamiana OX=4100 PE=2 SV=1	5
1::sp|cRAP109|P02788|TRFL_HUMAN	Lactotransferrin OS=Homo sapiens GN=LTF PE=1 SV=6	5
2::tr|A0A0F7JJ49|A0A0F7JJ49_NICBE	Glyceraldehyde-3-phosphate dehydrogenase OS=Nicotiana benthamiana OX=4100 GN=GAPC3 PE=2 SV=1	3
2::tr|A0A0F7JLU6|A0A0F7JLU6_NICBE	Glyceraldehyde-3-phosphate dehydrogenase OS=Nicotiana benthamiana OX=4100 GN=GAPC1 PE=2 SV=1	4
2::tr|A0A0A7HI84|A0A0A7HI84_NICBE	Histone H4 OS=Nicotiana benthamiana OX=4100 PE=2 SV=1	3
2::tr|A0ZVU2|A0ZVU2_NICBE	S-adenosylmethionine transporter OS=Nicotiana benthamiana OX=4100 GN=samt PE=2 SV=1	3

2::tr|I1Y996|I1Y996_NICBE	RabE1 OS=Nicotiana benthamiana OX=4100 PE=2 SV=1	2
1::sp|cRAP079|P61626|LYSC_HUMAN	Lysozyme C OS=Homo sapiens GN=LYZ PE=1 SV=1	2
2::tr|F8UV60|F8UV60_NICBE	Glutathione transferase OS=Nicotiana benthamiana OX=4100 GN=GSTU4 PE=2 SV=1	3
2::tr|A0A4D6IVV6|A0A4D6IVV6_NICBE	Stem-specific protein OS=Nicotiana benthamiana OX=4100 GN=LSP PE=4 SV=1	2
2::tr|F8WQS3|F8WQS3_NICBE	Ascorbate peroxidase (Fragment) OS=Nicotiana benthamiana OX=4100 GN=APX PE=2 SV=1	2
1::sp|cRAP013|P02662|CASA1_BOVIN	Alpha-S1-casein OS=Bos taurus GN=CSN1S1 PE=1 SV=2	2
1::sp|cRAP087|P02769|ALBU_BOVIN	Serum albumin OS=Bos taurus GN=ALB PE=1 SV=4	2
2::tr|A0A219V415|A0A219V415_NICBE	Histone H2B OS=Nicotiana benthamiana OX=4100 PE=2 SV=1	2
2::tr|Q6XX15|Q6XX15_NICBE	Glutathione S-transferase U3 (Fragment) OS=Nicotiana benthamiana OX=4100 PE=2 SV=1	2
2::tr|H9C954|H9C954_NICBE	Actin (Fragment) OS=Nicotiana benthamiana OX=4100 GN=act PE=2 SV=1	
2::tr|K7ZLE1|K7ZLE1_NICBE	Calcium-sensing receptor OS=Nicotiana benthamiana OX=4100 GN=NbCAS PE=2 SV=1	2
2::tr|E0X584|E0X584_NICBE	Harpin binding protein 1 (Fragment) OS=Nicotiana benthamiana OX=4100 GN=HBP1 PE=2 SV=1	1
1::sp|cRAP071|Q9NSB2|KRT84_HUMAN	Keratin, type II cuticular Hb4 OS=Homo sapiens GN=KRT84 PE=2 SV=2	2
2::tr|I0B7J3|I0B7J3_NICBE	Chloroplast PsbO3 OS=Nicotiana benthamiana OX=4100 GN=psbO3 PE=2 SV=1	2
2::tr|A0A0P0INT0|A0A0P0INT0_NICBE	Eukaryotic translation initiation factor 4A OS=Nicotiana benthamiana OX=4100 GN=eIF4A PE=2 SV=1	1
2::tr|A0A0F7R532|A0A0F7R532_NICBE	S-adenosylmethionine synthase OS=Nicotiana benthamiana OX=4100 GN=NbSAMS1a PE=2 SV=1	1
1::sp|cRAP_P12763|FETUA_BOVIN	Alpha-2-HS-glycoprotein OS=Bos taurus OX=9913 GN=AHSG PE=1 SV=2	1
2::tr|D2DWL6|D2DWL6_NICBE	QM OS=Nicotiana benthamiana OX=4100 PE=2 SV=1	2
2::tr|Q5EC25|Q5EC25_NICBE	Ubiquitin extension protein 1 OS=Nicotiana benthamiana OX=4100 GN=UEP1 PE=2 SV=1	2
2::tr|A0A1W5XFS7|A0A1W5XFS7_NICBE	HSP70 OS=Nicotiana benthamiana OX=4100 GN=HSP70 PE=2 SV=1	1

2::tr|F8WQS4|F8WQS4_NICBE	Quinone reductase (Fragment) OS=Nicotiana benthamiana OX=4100 GN=GR PE=2 SV=1	1
2::tr|I0B7J7|I0B7J7_NICBE	Chloroplast PsbP3 OS=Nicotiana benthamiana OX=4100 GN=psbP3 PE=2 SV=1	1
1::sp|cRAP_P22629|SAV_STRAV	Streptavidin OS=Streptomyces avidinii OX=1895 PE=1 SV=1	1
1::sp|cRAP035|P69905|HBA_HUMAN	Hemoglobin subunit alpha OS=Homo sapiens GN=HBA1 PE=1 SV=2	2
1::sp|cRAP006|P08758|ANXA5_HUMAN	Annexin A5 OS=Homo sapiens GN=ANXA5 PE=1 SV=2	1
2::sp|A0A0S4IJL0|RBS_NICBE	Ribulose bisphosphate carboxylase small chain, chloroplastic OS=Nicotiana benthamiana OX=4100 GN=rbcS PE=1 SV=1	1
1::sp|cRAP016|P02668|CASK_BOVIN	Kappa-casein OS=Bos taurus GN=CSN3 PE=1 SV=1	1
1::sp|cRAP095|Q06830|PRDX1_HUMAN	Peroxiredoxin-1 OS=Homo sapiens GN=PRDX1 PE=1 SV=1	1
2::tr|U6BM52|U6BM52_NICBE	Eukarytic translation initiation factor OS=Nicotiana benthamiana OX=4100 GN=eIF(iso)4E PE=2 SV=1	1
2::tr|A7IYM9|A7IYM9_NICBE	ADP-ribosylation factor 1 OS=Nicotiana benthamiana OX=4100 GN=ARF1 PE=2 SV=1	1
2::tr|Q6RI18|Q6RI18_NICBE	Isopentenyl/dimethylallyl diphosphate isomerase (Fragment) OS=Nicotiana benthamiana OX=4100 PE=2 SV=1	1
2::tr|R9W4N2|R9W4N2_NICBE	2-Cys peroxiredoxin (Fragment) OS=Nicotiana benthamiana OX=4100 PE=2 SV=1	1
2::tr|A0A387K109|A0A387K109_NICBE	GTP-binding nuclear protein OS=Nicotiana benthamiana OX=4100 GN=NbRan3 PE=2 SV=1	1
2::tr|C9DFB6|C9DFB6_NICBE	FAD-binding FR-type domain-containing protein (Fragment) OS=Nicotiana benthamiana OX=4100 PE=2 SV=1	1
2::tr|Q6XX17|Q6XX17_NICBE	Glutathione S-transferase U1 (Fragment) OS=Nicotiana benthamiana OX=4100 PE=2 SV=1	1

Table S4: 42aa-mCitrine sample protein hits.

Protein hit	Description	Matches
3::42aa_linker_mCitrine		627
1::sp|cRAP054|P04264|K2C1_HUMAN	Keratin, type II cytoskeletal 1 OS=Homo sapiens GN=KRT1 PE=1 SV=6	51
3::mCitrine		390
1::sp|cRAP041|P35527|K1C9_HUMAN	Keratin, type I cytoskeletal 9 OS=Homo sapiens GN=KRT9 PE=1 SV=3	45
1::sp|cRAP053|P35908|K22E_HUMAN	Keratin, type II cytoskeletal 2 epidermal OS=Homo sapiens GN=KRT2 PE=1 SV=2	33
1::sp|cRAP039|P13645|K1C10_HUMAN	Keratin, type I cytoskeletal 10 OS=Homo sapiens GN=KRT10 PE=1 SV=6	40
1::sp|cRAP032|P42212|GFP_AEQVI	Green fluorescent protein OS=Aequorea victoria GN=GFP PE=1 SV=1	278
2::tr|A0A0A8IBT8|A0A0A8IBT8_NICBE	Glyceraldehyde-3-phosphate dehydrogenase OS=Nicotiana benthamiana OX=4100 GN=NbGAPDH-A PE=2 SV=1	66
2::tr|E5LLE7|E5LLE7_NICBE	Phosphoglycerate kinase OS=Nicotiana benthamiana OX=4100 PE=2 SV=1	32
2::tr|A0A0F7JIC2|A0A0F7JIC2_NICBE	Glyceraldehyde-3-phosphate dehydrogenase OS=Nicotiana benthamiana OX=4100 GN=GAPC2 PE=2 SV=1	33
2::tr|A0A0F7JJ49|A0A0F7JJ49_NICBE	Glyceraldehyde-3-phosphate dehydrogenase OS=Nicotiana benthamiana OX=4100 GN=GAPC3 PE=2 SV=1	38
2::tr|I0B7J4|I0B7J4_NICBE	Chloroplast PsbO4 OS=Nicotiana benthamiana OX=4100 GN=psbO4 PE=2 SV=1	26
2::tr|A0A481NUV9|A0A481NUV9_NICBE	Chloroplast ferredoxin-NADP+ oxidoreductase (Fragment) OS=Nicotiana benthamiana OX=4100 PE=2 SV=1	31
2::tr|A0A173FEI6|A0A173FEI6_NICBE	ATP-dependent RNA helicase eIF4a OS=Nicotiana benthamiana OX=4100 GN=IF4a PE=2 SV=1	19
2::tr|I0B7J1|I0B7J1_NICBE	Chloroplast PsbO1 OS=Nicotiana benthamiana OX=4100 GN=psbO1 PE=2 SV=1	25
2::tr|A0A0F7JLU6|A0A0F7JLU6_NICBE	Glyceraldehyde-3-phosphate dehydrogenase OS=Nicotiana benthamiana OX=4100 GN=GAPC1 PE=2 SV=1	32
2::tr|Q58H58|Q58H58_NICBE	Chloroplast photosynthetic oxygen-evolving protein 33 kDa subunit	25

	OS=Nicotiana benthamiana OX=4100 GN=psbO PE=2 SV=1	
2::tr|H9A0F9|H9A0F9_NICBE	Eukaryotic initiation factor 4A-14 OS=Nicotiana benthamiana OX=4100 PE=3 SV=1	14
1::sp|cRAP112|P00761|TRYP_PIG	Trypsin OS=Sus scrofa PE=1 SV=1	63
2::tr|A0A0F7R532|A0A0F7R532_NICBE	S-adenosylmethionine synthase OS=Nicotiana benthamiana OX=4100 GN=NbSAMS1a PE=2 SV=1	17
2::tr|K7ZLE1|K7ZLE1_NICBE	Calcium-sensing receptor OS=Nicotiana benthamiana OX=4100 GN=NbCAS PE=2 SV=1	14
2::tr|Q45Q23|Q45Q23_NICBE	Prohibitin OS=Nicotiana benthamiana OX=4100 PE=2 SV=1	12
2::tr|A0A248QEL2|A0A248QEL2_NICBE	S-adenosylmethionine synthase OS=Nicotiana benthamiana OX=4100 GN=SAMs PE=2 SV=1	12
2::tr|Q153I4|Q153I4_NICBE	Nuclear pore complex protein RAE1b OS=Nicotiana benthamiana OX=4100 GN=RAE1 PE=2 SV=1	11
2::tr|W6JPQ9|W6JPQ9_NICBE	Nuclear pore complex protein RAE1a OS=Nicotiana benthamiana OX=4100 GN=NbRAE1a PE=2 SV=1	11
2::tr|H9C954|H9C954_NICBE	Actin (Fragment) OS=Nicotiana benthamiana OX=4100 GN=act PE=2 SV=1	12
2::tr|A0A286RNF7|A0A286RNF7_NICBE	Carbonic anhydrase OS=Nicotiana benthamiana OX=4100 PE=2 SV=1	15
2::tr|E1AXT8|E1AXT8_NICBE	Glycolate oxidase OS=Nicotiana benthamiana OX=4100 GN=GOX PE=1 SV=1	13
2::tr|A0A088F8F4|A0A088F8F4_NICBE	Chloroplast ATP-dependent Clp protease chaperone protein OS=Nicotiana benthamiana OX=4100 GN=ClpC1B PE=2 SV=1	10
2::tr|Q076B0|Q076B0_NICBE	RPN8 (Fragment) OS=Nicotiana benthamiana OX=4100 PE=2 SV=1	9
2::tr|A0A0H5AZB7|A0A0H5AZB7_NICBE	Farnesyl diphosphate synthase OS=Nicotiana benthamiana OX=4100 GN=NbFPPS1a PE=2 SV=1	7
2::tr|Q1EI36|Q1EI36_NICBE	Fibrillarin 2 OS=Nicotiana benthamiana OX=4100 GN=fib2 PE=2 SV=1	6
1::sp|cRAP040|O77727|K1C15_SHEEP	Keratin, type I cytoskeletal 15 OS=Ovis aries GN=KRT15 PE=2 SV=1	8
2::tr|A4D0J9|A4D0J9_NICBE	Carbonic anhydrase (Fragment) OS=Nicotiana benthamiana OX=4100 PE=2 SV=1	9
2::tr|Q2LAH0|Q2LAH0_NICBE	Chloroplast photosystem II 22 kDa component OS=Nicotiana benthamiana OX=4100 GN=psbS1 PE=2 SV=1	4
2::tr|Q2LAH1|Q2LAH1_NICBE	Chloroplast photosystem II 22 kDa component OS=Nicotiana benthamiana OX=4100 GN=psbS2 PE=4 SV=1	4

2::sp|A0A0S4IJL0|RBS_NICBE	Ribulose bisphosphate carboxylase small chain, chloroplastic OS=Nicotiana benthamiana OX=4100 GN=rbcS PE=1 SV=1	6
2::tr|A0A0F7QZM4|A0A0F7QZM4_NICBE	Aminocyclopropanecarboxylate oxidase OS=Nicotiana benthamiana OX=4100 GN=NbACO2a PE=2 SV=1	5
2::tr|A0A0G2RAA3|A0A0G2RAA3_NICBE	CK2 alpha subunit OS=Nicotiana benthamiana OX=4100 PE=2 SV=1	4
2::tr|B8R519|B8R519_NICBE	Ubiquitin (Fragment) OS=Nicotiana benthamiana OX=4100 PE=2 SV=1	4
2::tr|W6JJB0|W6JJB0_NICBE	Nuclear pore complex protein Seh1a OS=Nicotiana benthamiana OX=4100 GN=NbSeh1a PE=2 SV=1	4
2::tr|A0F0A7|A0F0A7_NICBE	Syntaxin (Fragment) OS=Nicotiana benthamiana OX=4100 GN=SYP132 PE=2 SV=1	4
2::tr|Q8SAQ3|Q8SAQ3_NICBE	Ribulose bisphosphate carboxylase small chain (Fragment) OS=Nicotiana benthamiana OX=4100 PE=2 SV=1	4
2::tr|Q076B1|Q076B1_NICBE	RPN9 (Fragment) OS=Nicotiana benthamiana OX=4100 PE=2 SV=1	5
2::tr|A0A219V415|A0A219V415_NICBE	Histone H2B OS=Nicotiana benthamiana OX=4100 PE=2 SV=1	4
2::tr|B8R6B6|B8R6B6_NICBE	ELI3 (Fragment) OS=Nicotiana benthamiana OX=4100 PE=2 SV=1	4
2::tr|Q5EC25|Q5EC25_NICBE	Ubiquitin extension protein 1 OS=Nicotiana benthamiana OX=4100 GN=UEP1 PE=2 SV=1	4
2::tr|C5MTW1|C5MTW1_NICBE	Heterotrimeric G protein beta 1 subunit OS=Nicotiana benthamiana OX=4100 PE=2 SV=1	4
2::tr|A0A1S5WM37|A0A1S5WM37_NICBE	Papain-like cysteine proteinase 6 OS=Nicotiana benthamiana OX=4100 PE=2 SV=1	3
2::tr|A0A455QYV0|A0A455QYV0_NICBE	Receptor for activated C kinase 1 OS=Nicotiana benthamiana OX=4100 GN=NbRACK1 PE=2 SV=1	5
2::tr|A0A1X9RIL3|A0A1X9RIL3_NICBE	S-adenosylmethionine synthase OS=Nicotiana benthamiana OX=4100 GN=SAMs1 PE=2 SV=1	4
2::tr|A0A0H5BGR5|A0A0H5BGR5_NICBE	Acetoacetyl-coenzyme A thiolase OS=Nicotiana benthamiana OX=4100 GN=NbACAT1a PE=2 SV=1	4
2::tr|Q6XX19|Q6XX19_NICBE	Translation elongation factor 1 alpha (Fragment) OS=Nicotiana benthamiana OX=4100 PE=2 SV=1	4
2::tr|W6JJ90|W6JJ90_NICBE	Nuclear pore complex protein Sec13d OS=Nicotiana benthamiana OX=4100 GN=NbSec13d PE=2 SV=1	2
1::sp|cRAP013|P02662|CASA1_BOVIN	Alpha-S1-casein OS=Bos taurus GN=CSN1S1 PE=1 SV=2	3
2::tr|E0X584|E0X584_NICBE	Harpin binding protein 1 (Fragment) OS=Nicotiana benthamiana OX=4100 GN=HBP1 PE=2 SV=1	2

2::tr|Q84V57|Q84V57_NICBE	Pectinesterase OS=Nicotiana benthamiana OX=4100 PE=2 SV=1	2
2::tr|A0A1W5XFS7|A0A1W5XFS7_NICBE	HSP70 OS=Nicotiana benthamiana OX=4100 GN=HSP70 PE=2 SV=1	3
2::tr|J7EUE8|J7EUE8_NICBE	Germin-like protein OS=Nicotiana benthamiana OX=4100 PE=2 SV=1	2
2::tr|Q8H1A1|Q8H1A1_NICBE	Actin (Fragment) OS=Nicotiana benthamiana OX=4100 GN=actin PE=3 SV=1	2
2::tr|A0ZVU2|A0ZVU2_NICBE	S-adenosylmethionine transporter OS=Nicotiana benthamiana OX=4100 GN=samt PE=2 SV=1	2
2::tr|T1VY78|T1VY78_NICBE	Epoxide hydrolase (Fragment) OS=Nicotiana benthamiana OX=4100 GN=EH1.1 PE=2 SV=1	3
2::tr|Q45Q24|Q45Q24_NICBE	Prohibitin OS=Nicotiana benthamiana OX=4100 PE=2 SV=1	3
2::tr|A0A0E3JCP4|A0A0E3JCP4_NICBE	Developmentally-regulated plasma membrane polypeptide OS=Nicotiana benthamiana OX=4100 GN=DREPP PE=2 SV=1	2
1::sp|cRAP071|Q9NSB2|KRT84_HUMAN	Keratin, type II cuticular Hb4 OS=Homo sapiens GN=KRT84 PE=2 SV=2	3
2::tr|Q5XPZ0|Q5XPZ0_NICBE	Adenosine kinase (Fragment) OS=Nicotiana benthamiana OX=4100 GN=ADK PE=2 SV=1	2
2::tr|Q1MSG9|Q1MSG9_NICBE	ALY protein OS=Nicotiana benthamiana OX=4100 GN=aly PE=2 SV=1	2
2::tr|D5LT98|D5LT98_NICBE	Chloroplast elongation factor TuB (Fragment) OS=Nicotiana benthamiana OX=4100 GN=CpEF-TuB PE=2 SV=1	3
2::tr|A0A0A8K9V3|A0A0A8K9V3_NICBE	Geranylgeranyl reductase OS=Nicotiana benthamiana OX=4100 GN=chlP PE=2 SV=1	4
2::tr|I1Y996|I1Y996_NICBE	RabE1 OS=Nicotiana benthamiana OX=4100 PE=2 SV=1	3
2::tr|Q8H0B4|Q8H0B4_NICBE	Mitogen-activated protein kinase OS=Nicotiana benthamiana OX=4100 GN=NbWIPK PE=2 SV=1	2
2::tr|B6RFK8|B6RFK8_NICBE	Branched-chain-amino-acid aminotransferase OS=Nicotiana benthamiana OX=4100 GN=BCAT PE=2 SV=1	2
2::tr|A7L4B4|A7L4B4_NICBE	Histone H3 OS=Nicotiana benthamiana OX=4100 PE=2 SV=1	2
2::tr|A0A0A7HI84|A0A0A7HI84_NICBE	Histone H4 OS=Nicotiana benthamiana OX=4100 PE=2 SV=1	2
2::tr|D2DWL6|D2DWL6_NICBE	QM OS=Nicotiana benthamiana OX=4100 PE=2 SV=1	1
2::tr|B7U9Z3|B7U9Z3_NICBE	ER luminal-binding protein OS=Nicotiana benthamiana OX=4100 GN=BLP4 PE=2 SV=1	2

2::tr|H9A0F8|H9A0F8_NICBE	Brix domain-containing protein OS=Nicotiana benthamiana OX=4100 PE=4 SV=1	1
2::tr|A0A5B8NGM1|A0A5B8NGM1_NICBE	Arogenate dehydratase OS=Nicotiana benthamiana OX=4100 PE=2 SV=1	2
1::sp|cRAP002|P02768|ALBU_HUMAN	Serum albumin OS=Homo sapiens GN=ALB PE=1 SV=2	1
2::tr|A0A384E148|A0A384E148_NICBE	Alpha-galactosidase OS=Nicotiana benthamiana OX=4100 PE=1 SV=1	1
2::tr|A0A0M3SBS3|A0A0M3SBS3_NICBE	Heat shock protein 90-3 OS=Nicotiana benthamiana OX=4100 PE=2 SV=1	1
2::tr|A7IYM9|A7IYM9_NICBE	ADP-ribosylation factor 1 OS=Nicotiana benthamiana OX=4100 GN=ARF1 PE=2 SV=1	1
1::sp|cRAP015|P02666|CASB_BOVIN	Beta-casein OS=Bos taurus GN=CSN2 PE=1 SV=2	2
2::tr|U3SXQ9|U3SXQ9_NICBE	Translationally controlled tumor protein OS=Nicotiana benthamiana OX=4100 GN=NbTCTP PE=2 SV=1	1
1::sp|cRAP018|P07339|CATD_HUMAN	Cathepsin D OS=Homo sapiens GN=CTSD PE=1 SV=1	1
2::tr|A5JGX6|A5JGX6_NICBE	RAN GTPase-activating protein 2 OS=Nicotiana benthamiana OX=4100 GN=RanGAP2 PE=2 SV=1	1
2::tr|A0A060INU6|A0A060INU6_NICBE	Acyl-[acyl-carrier-protein] desaturase OS=Nicotiana benthamiana OX=4100 PE=2 SV=1	1
1::sp|cRAP079|P61626|LYSC_HUMAN	Lysozyme C OS=Homo sapiens GN=LYZ PE=1 SV=1	1
2::tr|A0A1P8SF00|A0A1P8SF00_NICBE	RPM1-interacting protein 4 OS=Nicotiana benthamiana OX=4100 GN=RIN4 PE=2 SV=1	1
2::tr|W6JLF3|W6JLF3_NICBE	Nuclear pore complex protein gp210a OS=Nicotiana benthamiana OX=4100 GN=Nbgp210a PE=2 SV=1	1
2::tr|W6JLF6|W6JLF6_NICBE	Nuclear pore complex protein NUP35 OS=Nicotiana benthamiana OX=4100 GN=NbNup35b PE=2 SV=1	1
1::sp|cRAP016|P02668|CASK_BOVIN	Kappa-casein OS=Bos taurus GN=CSN3 PE=1 SV=1	1
1::sp|cRAP094|P62937|PPIA_HUMAN	Peptidyl-prolyl cis-trans isomerase A OS=Homo sapiens GN=PPIA PE=1 SV=2	1
2::sp|P53800|FDFT_NICBE	Squalene synthase OS=Nicotiana benthamiana OX=4100 PE=2 SV=1	1
2::sp|Q84N38|PVIP_NICBE	OBERON-like protein OS=Nicotiana benthamiana OX=4100 GN=PVIP PE=1 SV=1	1
2::tr|A0A0S0N5Y9|A0A0S0N5Y9_NICBE	Tubulin alpha chain OS=Nicotiana benthamiana OX=4100 GN=TUA6 PE=2 SV=1	1
2::tr|A0A4D6IA34|A0A4D6IA34_NICBE	Chloroplastic PsbP1 OS=Nicotiana benthamiana OX=4100 PE=2 SV=1	1

2::tr|R9W4N2|R9W4N2_NICBE	2-Cys peroxiredoxin (Fragment) OS=Nicotiana benthamiana OX=4100 PE=2 SV=1	1
2::tr|Q0WYB7|Q0WYB7_NICBE	Dirigent protein OS=Nicotiana benthamiana OX=4100 PE=2 SV=1	1
2::tr|Q58IU5|Q58IU5_NICBE	Non-intrinsic ABC protein (Fragment) OS=Nicotiana benthamiana OX=4100 PE=2 SV=1	1
2::tr|K0IBB4|K0IBB4_NICBE	Catalase (Fragment) OS=Nicotiana benthamiana OX=4100 PE=2 SV=1	1
2::tr|I0B7J9|I0B7J9_NICBE	Chloroplast PsbQ1 OS=Nicotiana benthamiana OX=4100 GN=psbQ1 PE=2 SV=1	1
2::tr|A0A0S3ANE0|A0A0S3ANE0_NICBE	NRC2b OS=Nicotiana benthamiana OX=4100 PE=2 SV=1	1

Data S1. (separate file)
Data S1: Fasta file of vector and coding sequences mentioned in this study
